# Predictive Value of Lymphocyte‐to‐Neutrophil Ratio and Platelet‐to‐Neutrophil Ratio on PD‐L1 Expression in Lung Cancer

**DOI:** 10.1111/crj.13821

**Published:** 2024-08-14

**Authors:** Shun‐Shun Cui, Ya Shen, Rui‐Qing Yang

**Affiliations:** ^1^ Department of Respiratory and Critical Care Medicine Fuyang People's Hospital Fuyang China; ^2^ Department of Respiratory and Critical Care Medicine Fuyang Infectious Disease Clinical College of Anhui Medical University Fuyang China

**Keywords:** lung cancer, lymphocyte‐to‐neutrophil ratio, platelet‐to‐neutrophil ratio, programmed death receptor ligand 1

## Abstract

**Objective:**

This study aimed to examine the predictive effect of the lymphocyte‐to‐neutrophil ratio (LNR) and the platelet‐to‐neutrophil ratio (PNR) on the expression of programmed death receptor ligand 1 (PD‐L1) in patients diagnosed with lung cancer.

**Methods:**

The clinical records of 86 patients diagnosed with lung cancer between January 2020 and February 2022 at Fu Yang People's Hospital were retrospectively analyzed. The records included information on age, gender, smoking history, hematological indices at the time of admission, staging of the lung malignancy, histopathological subtype, comorbidities, and the expression levels of PD‐L1. Patients were stratified into two distinct cohorts based on their PD‐L1 expression levels: Those with an expression level greater than or equal to 1% were classified into the PD‐L1 positive expression group, while the remainder were categorized as the PD‐L1 negative expression group. Univariate analysis and multivariate logistic regression analysis were used to identify the influencing factors of PD‐L1, and the diagnostic efficacy was calculated using the receiver operating characteristic (ROC) curve.

**Results:**

Upon analysis, the PD‐L1 positive expression group manifested notably lower values as compared to their counterparts in the PD‐L1 negative expression group (LNR: 0.262 ± 0.105 vs. 0.390 ± 0.201; PNR: 41.03 [29.64, 50.11] vs. 49.50 [37.38, 73.83]), and these differences were statistically significant. There was a notable disparity in PD‐L1 expression based on gender, with males exhibiting a statistically significant higher positivity rate compared to females. Furthermore, patients in Stages I–III of the disease demonstrated a markedly elevated PD‐L1 positivity rate compared to those in Stage IV (*p* < 0.05). Incorporating univariates with statistical differences into multivariate logistic regression analysis suggests that stage and LNR are independent risk factors for PD‐L1 negative expression. ROC curve analyses revealed that the area under the ROC curve (AUC) for LNR as an indicator for PD‐L1 positive expression stood at 0.706, while the AUC for PNR was calculated at 0.687.

**Conclusion:**

PD‐L1 expression is correlated with gender and lung cancer staging, and LNR and PNR have a predictive value for PD‐L1 expression.

## Graphical abstract

This study investigated the predictive value of LNR and PNR on PD‐L1 expression in lung cancer patients. Clinical records of 86 patients were analyzed, revealing a significant association between gender and PD‐L1 expression. Patients in Stages I–III had higher PD‐L1 positivity rates compared to Stage IV. LNR and PNR were found to be predictive indicators of PD‐L1 expression.

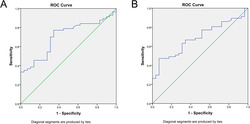

**Abbreviations**
AUC: area under the curve
ICIs: immune checkpoint inhibitors
IL‐1: interleukin‐1
IL‐6: interleukin‐6
LNR: lymphocyte‐to‐neutrophil ratio
PD‐1: programmed death protein 1
PD‐L1: programmed death receptor ligand 1
PNR: platelet‐to‐neutrophil ratio
ROC: receiver operating characteristic
TMB: tumor mutational burden

## 1 Introduction

Based on the GLOBOCAN 2020 statistical report, lung cancer remains the predominant cause of cancer‐related mortalities worldwide. Notably, among the malignant neoplasms in China, it exhibits the highest incidence and mortality, suggesting an overall adverse prognosis [1]. Over time, several therapeutic paradigms have evolved. Immunotherapy, distinct from the traditional chemoradiotherapy and targeted therapies, has garnered substantial attention in contemporary oncological research. Yet, studies have indicated that the efficacy of immunotherapy, in the absence of gene detection, stands at a mere 20% [2]. Given the economic implications of immunotherapy, identifying a cost‐effective biomarker that can reliably predict the therapeutic outcome is imperative. Current studies recognize associations between the efficacy of immunotherapy and factors such as programmed death protein 1 (PD‐1), its ligand PD‐L1, tumor mutational burden (TMB), and microsatellite instability. Although PD‐L1 expression assays are extensively used clinically to guide immunotherapeutic approaches, these evaluations necessitate specific tissue specimens, leading to elevated costs and prolonged processing times. Hence, the oncology community is in pursuit of a financially viable and readily available biomarker. Emerging evidence suggests a linkage between tumor genesis and progression with chronic inflammation. Further, the inflammatory mediators of the tumor microenvironment have demonstrated capabilities to modulate PD‐L1 expression through multiple mechanisms [3]. Common hematological parameters, including neutrophils, lymphocytes, platelets, and their respective ratios, can be indicative of the systemic inflammatory status.

The primary objective of our study was to ascertain the prognostic capabilities of these routine hematological markers, as well as their ratios, in predicting PD‐L1 expression.

## 2 Materials and Methods

### 2.1 Participants

In a retrospective study conducted at Fu Yang People's Hospital, the medical records of 86 patients diagnosed with lung cancer from January 2020 to February 2022 were meticulously examined.

The following were the inclusion criteria:

1. A definitive lung cancer diagnosis confirmed through histopathological assessment.
2. Availability of pathological biopsy specimens that met the standards for PD‐L1 evaluation, adhering to the guidelines and standards set forth in expert consensus [4].
3. Comprehensive and intact medical documentation.

The exclusion criteria were as follows:

1. Comorbidities involving multiple malignancies.
2. Presence of severe infections or intake of glucocorticoids or granulocyte colony‐stimulating factor.
3. Hematological abnormalities that could potentially alter the complete blood count outcomes.
4. Cases where PD‐L1 testing was unfeasible or where the diagnosis was rendered at an external institution without the accompanying pathological specimen.
5. Insufficient data or significant aberrations in the complete blood count, attributed to extraneous factors.

### 2.2 Research Methods

Patient data were meticulously collected, including age, gender, smoking history, stage, pathological type, and comorbidities. Furthermore, results from PD‐L1 testing and concurrent hematological indices—including counts for neutrophils, lymphocytes, monocytes, eosinophils, and platelets—were recorded. Derived metrics, such as the lymphocyte‐to‐neutrophil ratio (LNR) and platelet‐to‐neutrophil ratio (PNR), were subsequently computed.

### 2.3 Statistical Methods

Statistical evaluations were performed with the SPSS 22.0 software. The SPSS 22.0 software was used for data analysis, and quantitative data that followed normal distribution and homogeneity of variance were described using mean ± standard deviation (*x* ± *s*). Two independent‐sample *t*‐tests were used for intergroup comparison; quantitative data that do not follow a normal distribution are described using median and interquartile intervals, *M* (Q1, Q3), and nonparametric tests are used for intergroup comparison. A frequency description is used for qualitative data, and a chi‐square test is used for intergroup comparison. Variables with significant differences in univariate analysis were selected to enter the multivariate analysis model, and forward stepwise regression was used to perform logistic regression analysis on the risk factors for disease onset. The receiver operating characteristic (ROC) curve was used to calculate the area under the curve (AUC): AUC ≤ 0.5, with no diagnostic value; 0.5 < AUC < 0.7, with low predictive diagnostic value; 0.7 ≤ AUC < 0.9, with moderate predictive diagnostic value; AUC ≥ 0.9, with high predictive diagnostic value [5]. The difference is statistically significant with *p* < 0.05.

## 3 Results

### 3.1 Relationship Between PD‐L1 Expression and Age, Gender, Smoking History, Stage, Pathological Type, and Comorbidities

In this study, 86 patients diagnosed with lung cancer were analyzed. Each patient underwent PD‐L1 testing, revealing 29 positive cases and 57 negative cases, equating to a positive expression rate of 33.7%. Notably, the rate of PD‐L1 expression in male participants was significantly higher in comparison to that in female participants. Additionally, PD‐L1 expression was markedly higher in Stages I–III compared to Stage IV (*p* < 0.05). However, no significant associations were found between PD‐L1 expression and factors such as age, smoking history, pathological subtype, or comorbidities. Comprehensive details are presented in Table 1.

**TABLE 1:** Relationship between PD‐L1 expression and age, gender, smoking history, cancer stage, pathological type, and comorbidities.

Indicator			PD‐L1 positive (*n* = 29)	PD‐L1 negative (*n* = 57)	*χ* ^2^/*t* value	*p*
Age (years)	≥ 60		22	35	1.798	0.180
	< 60		7	22		
Gender	Male		27	40	5.871	0.015
	Female		2	17		
Smoking history	Yes		21	30	3.117	0.077
	No		8	27		
Staging	I–III stages		19	22	5.584	0.018
	IV stage		10	35		
Pathological type	Lung glandular cancer		11	32	2.549	0.110
	Other types		18	25		
Comorbidities	Hypertension	Yes	11	23	0.047	0.828
		No	18	34		
	Diabetes	Yes	6	6	0.915	0.339
		No	23	51		
	Coronary heart disease	Yes	3	7	0.000	1.000
		No	26	50		
	Cerebral infarction	Yes	6	3	3.374	0.066
		No	23	54		

### 3.2 Relationship Between PD‐L1 Expression and Neutrophil Count, Lymphocyte Count, Monocyte Count, Eosinophil Count, Platelet Count, LNR, and PNR

In comparing the PD‐L1 positive expression group with the PD‐L1 negative expression group, no statistically significant differences were observed in parameters such as lymphocyte, monocyte, eosinophil, and platelet counts. Nevertheless, the PD‐L1 negative expression group demonstrated a significantly reduced neutrophil count (5.340 [4.215, 6.775] vs. 4.160 [3.160, 5.770]), LNR (0.262 ± 0.105 vs. 0.390 ± 0.201) and PNR (41.03 [29.64, 50.11] vs. 49.50 [37.38, 73.83]). Detailed data are provided in Table 2. Multivariate logistic regression analysis was performed on indicators with statistical differences, indicating that stage and LNR are independent risk factors for PD‐L1 negative expression (Table 3).

**TABLE 2:** Relationship between PD‐L1 expression and white blood cell, neutrophil, lymphocyte, monocyte, eosinophil, platelet, LNR, and PNR counts.

	PD‐L1 positive expression	PD‐L1 negative expression	*t*/*z* value	*p*
WBC	7.510 (6.215, 9.310)	6.600 (5.230, 8.175)	−1.864	0.062
Neutrophil count	5.340 (4.215, 6.775)	4.160 (3.160, 5.770)	−2.553	0.011
Lymphocyte count	1.395 ± 0.523	1.586 ± 0.558	−1.536	0.128
Monocyte count	0.590 (0.440, 0.710)	0.460 (0.345, 0.625)	−1.901	0.057
Eosinophil count	0.160 (0.095, 0.330)	0.150 (0.050, 0.215)	−1.476	0.140
Platelet count	211 (163, 305)	220 (178, 281)	−0.808	0.419
LNR	0.262 ± 0.105	0.390 ± 0.201	−3.868	< 0.001
PNR	41.03 (29.64, 50.11)	49.50 (37.38, 73.83)	−2.823	0.005

Abbreviations: LNR: lymphocyte‐to‐neutrophil ratio; PNR: platelet‐to‐neutrophil ratio.

**TABLE 3:** Multivariate logistic regression analysis of PD‐L1 expression.

Index	RC	SE	Wald	*p*	OR	95% CI
LNR	5.512	1.874	8.648	0.003	247.5	6.3–9747.9
Staging	1.371	0.523	6.871	0.009	3.9	1.4–11.0
Constant term	−1.731	0.684	6.397	0.011	0.2	

### 3.3 The Predictive Value of LNR and PNR in PD‐L1 Expression

**FIGURE 1:**
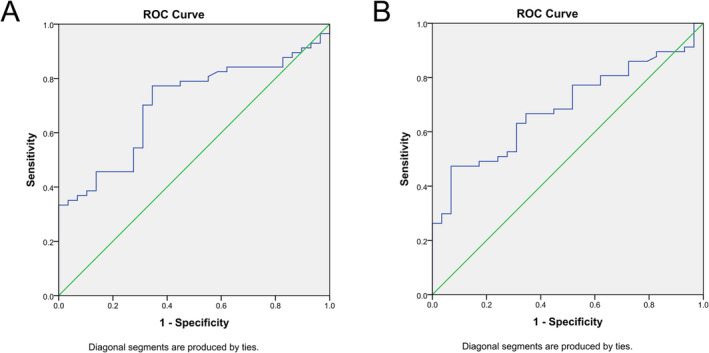
(A) ROC curve of the lymphocyte‐to‐neutrophil ratio (LNR) between PD‐L1 positive and negative expression groups. *Note:* The area under the ROC curve (AUC) of LNR as an indicator for PD‐L1 positive expression was 0.706 (*p* = 0.002); when the diagnostic cutoff value was set at 0.2644, the highest Youden index was 1.427, the sensitivity was 77.2%, and the specificity was 65.5%. (B) ROC curve of the platelet‐to‐neutrophil ratio (PNR) between PD‐L1 positive and negative expression groups. *Note:* The area under the ROC curve (AUC) of PNR as an indicator for PD‐L1 positive expression was 0.687 (*p* = 0.005); when the diagnostic cutoff value was 53.8954, the highest Youden index was 1.405, the sensitivity was 47.4%, and the specificity was 93.1%.

The ROC curve was constructed for patients exhibiting PD‐L1 positive expression using LNR and PNR, neutrophil count as variables. The AUC of PD‐L1 expression positive for neutrophil count is 0.331, which has no predictive value. The AUC for LNR as a determinant of PD‐L1 positive expression was 0.706 (*p* = 0.002). A diagnostic threshold of 0.2644 was employed, and the optimal Youden index was calculated as 1.427, with a sensitivity of 77.2% and a specificity of 65.5% (refer to Figure 1A). Similarly, the AUC for PNR in assessing PD‐L1 positive expression was ascertained to be 0.687 (*p* = 0.005). Upon setting the diagnostic cutoff value at 53.8954, the peak Youden index reached 1.405, accompanied by a sensitivity of 47.4% and a specificity of 93.1% (refer to Figure 1B).

## 4 Discussion

Lung cancer, a malignancy originating from the bronchial mucosal epithelium and associated lung tissues, consistently presents with elevated morbidity and mortality rates. Contemporary treatment modalities primarily encompass surgical intervention, chemoradiotherapy, targeted treatments, and antivascular therapy. However, prognosis remains poor for the majority, and the 5‐year survival rate is notably low. In recent academic studies, immune checkpoint inhibitors (ICIs) have garnered substantial attention and have been subsequently approved for treating diverse tumors, including malignant melanoma, lung cancer, and urothelial carcinoma [5, 6]. Currently, the therapeutic effect of ICIs is mainly assessed based on the expression of PD‐L1. Both PD‐L1 and PD‐L2 function as principal ligands for PD‐1. Upon the conjugation of PD‐L1 with PD‐1, immune signals are transmitted, leading to the inhibition of T‐cell activation and proliferation—a mechanism implicated in tumor immune evasion [7]. Prior studies have recorded PD‐L1 expression positivity rates ranging from 39.9% to 53.1% [8]. Concurrently, studies conducted in China, including those by Gao et al., indicated a higher positivity rate at 61.67% [9]. Based on the results of our research, the PD‐L1 positivity rate was 33.7% (29/86), which appears comparatively diminished. Our findings suggest a correlation between PD‐L1 positive expression and both gender and tumor stage, revealing heightened positivity rates in males than in females and more in Stages I–III than in Stage IV. These observations diverge from preceding studies, such as those led by Jiang et al. and Ma and Ma, where gender did not seem to influence PD‐L1 expression, though tumor stage evidently did [10, 11]. It is imperative to acknowledge that their stage groupings were not congruent with ours. Such disparities in outcomes might arise from diverse population selections in different studies. Numerous pertinent Chinese studies are characterized by their single‐center nature and relatively small sample cohorts. Our research predominantly hinged on biopsy specimens, with a dearth of surgically acquired samples—a factor that might influence the experimental findings. Given these caveats, future research endeavors could benefit from multicenter collaborations involving more expansive sample populations to verify these experimental results.

The result of a prior study indicates that the genesis and progression of tumors are intricately linked with inflammation [12, 13]. Activated inflammatory cells and the ensuing inflammatory mediators can stimulate the creation of new blood and lymphatic vessels. This creates a conducive tumor microenvironment that fosters the growth and differentiation of nascent tumors. Advanced tumor‐associated inflammation compromises immune functionality, facilitating tumor immune evasion and metastasis. Subsequent studies have ascertained that inflammatory mediators within the tumor microenvironment can modulate PD‐L1 levels either directly or indirectly through relevant signaling pathways and transcription factors [14]. Hematological indicators largely mirror systemic inflammation, suggesting their potential utility as predictors of PD‐L1 expression. Neutrophils, the most abundant cells in peripheral blood, signify heightened inflammatory responses when elevated. Elevated neutrophil levels can both promote inflammatory mediator release, such as IL‐1 and IL‐6, fostering tumor angiogenesis and metastasis, and inhibit lymphocytes and natural killer cells, enhancing tumor immune evasion capabilities. As primary immune cells, lymphocytes chiefly act in immune surveillance, curbing tumor proliferation and metastasis. Reduced lymphocyte counts diminish the antitumor immune efficacy of the body, paving the way for tumor advancement. By secreting cytokines and chemokines, platelets establish a hypercoagulable state, potentially abetting tumor cell proliferation, invasion, and metastasis. An elevated platelet count has been associated with unfavorable tumor prognoses [15, 16, 17]. Previous findings have underscored that a high neutrophil count, diminished lymphocyte count, and elevated platelet count could correlate with suboptimal responses to immunotherapy [18]. Currently, PD‐L1 stands as a primary predictive marker for gauging immunotherapy efficacy in clinical settings. PD‐L1 positive expression typically aligns with enhanced therapeutic outcomes compared to negative expression. In this study, the LNR in the PD‐L1 positive expression group was significantly lower than in the PD‐L1 negative expression group, implying reduced lymphocyte counts and/or increased neutrophil counts in those with PD‐L1 positive expression. Such observations are in line with prior findings. The ROC curve of the LNR yielded an AUC of 0.706, signifying a high predictive capability for PD‐L1 expression. Notably, the PNR in the PD‐L1 positive expression group was appreciably lower than in its negative counterpart. While the ROC curve of the PNR indicated predictive relevance for PD‐L1 expression, deeper exploration into the relationship between the two cohorts is required. In this study, differences in lymphocyte, and platelet counts between the PD‐L1 positive expression and negative expression groups were not statistically significant. This could be attributed to the ubiquity in peripheral blood and susceptibility to systemic influences of these cells. However, the related ratios (LNR and PNR) may offer a more nuanced perspective, potentially negating these systemic impacts. Given the retrospective nature and having been conducted at a single center with a limited sample size, the findings of this study may bear inherent biases.

To conclude, PD‐L1 expression is notably elevated in male patients and those diagnosed with Stage I–III lung cancer. Furthermore, the LNR and PNR in peripheral blood present significant predictive potential for PD‐L1 expression.

## Author Contributions

Conception and design of the research: Shun‐Shun Cui and Rui‐Qing Yang. Acquisition of data: Shun‐Shun Cui. Analysis and interpretation of the data: Shun‐Shun Cui and Ya Shen. Statistical analysis: Shun‐Shun Cui and Ya Shen. Writing of the manuscript: Shun‐Shun Cui. Critical revision of the manuscript for intellectual content: Shun‐Shun Cui and Rui‐Qing Yang. All authors read and approved the final draft.

## Ethics Statement

This study was conducted with approval from the Ethics Committee of Fuyang People's Hospital. This study was conducted in accordance with the Declaration of Helsinki. Written informed consent was obtained from all participants.

## Conflicts of Interest

The authors declare no conflicts of interest.

## Data Availability

The datasets used and/or analyzed during the current study are available from the corresponding author on reasonable request. We declare that materials described in the manuscript, including all relevant raw data, will be freely available to any scientist wishing to use them for non‐commercial purposes, without breaching participant confidentiality.
